# IgE to Galactose‐α‐1,3‐Galactose (α‐Gal) and Relevance to Ustekinumab First‐Dose Infusion Reactions and Allergenicity

**DOI:** 10.1002/jgh3.70374

**Published:** 2026-02-24

**Authors:** Preethi G. Venkat, Jill F. Nehrbas, Pamela Schoppee Bortz, Thomas A. Platts‐Mills, Brian W. Behm, Esteban J. Figueroa, Jeffrey M. Wilson

**Affiliations:** ^1^ Division of Gastroenterology and Hepatology University of Virginia Charlottesville Virginia USA; ^2^ Division of Allergy and Clinical Immunology University of Virginia Charlottesville Virginia USA

**Keywords:** allergenicity, alpha‐gal, infliximab, infusion reaction, ustekinumab

## Abstract

**Aims:**

Ustekinumab, a monoclonal antibody (mAb) used to treat inflammatory bowel disease, has been associated with immediate hypersensitivity reactions during first intravenous infusion. While rare, the mechanism for these reactions is unclear. IgE to the oligosaccharide galactose‐α‐1,3‐galactose (α‐gal) is a cause of allergy to mammalian meat and other mammalian products that express α‐gal. Given that ustekinumab (Stelara) is produced in murine SP2/0 cells, a cell line known to introduce α‐gal during post‐translational modification, we hypothesized that α‐gal could explain some of these reactions.

**Methods and Results:**

We describe six patients with Crohn's disease who experienced acute reactions during ustekinumab infusion and were found to have IgE specific to α‐gal. Serum or cells from a separate group of α‐gal IgE‐sensitized patients with red meat allergy were used for in vitro experiments to investigate ustekinumab allergenicity. ImmunoCAP binding assays confirmed that IgE from four red meat allergic patients, but not controls, bound to ustekinumab on the solid phase. This binding was inhibited by the α‐gal‐containing glycoprotein beef thyroglobulin. Basophil activation tests (BAT), carried out to assess functional activity, revealed dose‐dependent activation with ustekinumab from four α‐gal‐sensitized individuals, but not controls. Two other mAbs produced in SP2/0 cells (cetuximab and infliximab) as well as a representative mAb produced in Chinese Hamster Ovary (CHO) cells (vedolizumab) were evaluated as comparators, with the mAbs from SP2/0 but not CHO cells eliciting BAT activity.

**Conclusions:**

Pre‐existing IgE to α‐gal may account for some first‐dose infusion reactions to ustekinumab. Screening for α‐gal sensitization in patients with alpha‐gal syndrome should be considered prior to ustekinumab infusion.

## 1 Background

Ustekinumab is a humanized monoclonal antibody (mAb) targeting the p40 subunit shared by interleukins 12 and 23. It is FDA‐approved for treating inflammatory bowel disease (IBD), as well as psoriasis and psoriatic arthritis. While the prescribing information for ustekinumab (Stelara, Janssen Pharmaceuticals, Beerse, Belgium) reports a rare hypersensitivity reaction to intravenous infusion (0.08%), IBD trials have observed higher rates ranging from 0.9% to 4.5% [1, 2, 3, 4, 5]. Notably, real‐world data show 93% of patients who experience infusion reactions can safely continue treatment with subcutaneous injections [6].

The mechanism underlying these reactions remains unclear, as IgE‐mediated hypersensitivity reactions typically require prior exposure to the drug. One potential explanation involves galactose‐α‐1,3‐galactose (α‐gal), a glycan implicated in mammalian meat allergy and first‐dose anaphylactic reactions to the mAb cetuximab [7, 8, 9]. Severe hypersensitivity reactions during the initial infusion of cetuximab, a chimeric mAb targeting the epidermal growth factor receptor (EGFR) used in cancer treatment, have been causally linked to pre‐existing IgE antibodies specific to α‐gal [9, 10, 11]. These antibodies are best understood to result from tick bites. In the United States, the lone star tick ( *Amblyomma americanum* ) is most strongly linked with α‐gal sensitization but other species of ticks contribute to α‐gal sensitization outside of North America [12, 13, 14]. Such patients who develop IgE to α‐gal and experience allergic reactions to mammalian meat or other mammalian‐derived products fit criteria for the alpha‐gal syndrome (AGS). Of note, it is increasingly appreciated that not all individuals who have IgE to α‐gal develop allergy to mammalian meat [15]. While urticaria is classically associated with AGS, it is increasingly recognized that the syndrome can also present with isolated gastrointestinal symptoms [16, 17].

The reason cetuximab is thought to have abundant α‐gal relates to its production in the mouse myeloma SP2/0 cell line, which introduces α‐gal glycans on the Fab domain during post‐translational modification [9, 18]. Infliximab, another mAb produced in the murine SP2/0 cell line, is also associated with rare cases of α‐gal‐related hypersensitivity reactions [19, 20]. By contrast, most biologics generated in Chinese Hamster Ovary (CHO) cells are not thought to express appreciable α‐gal and likely do not represent significant risk for alpha‐gal sensitized patients [9, 18]. Given its production in the murine SP2/0 cell line, we hypothesized that ustekinumab expresses functionally relevant α‐gal and that pre‐existing IgE to α‐gal could explain some first‐dose infusion reactions. This hypothesis was borne, in part, from investigation of cases of first infusion reactions to ustekinumab in our clinical practice. Here, we describe features of α‐gal sensitized patients who reacted to ustekinumab from an area of the United States where lone star ticks are endemic and the α‐gal red meat allergy is common. We also carried out in vitro testing to assess α‐gal‐related allergenicity of ustekinumab and other biologics.

## 2 Methods

Herein, we describe a mixed‐methods study that includes description of a series of cases seen at the University of Virginia (UVA) with first infusion reactions to ustekinumab, followed by in vitro investigation into ustekinumab allergenicity. All aspects of this study involved ustekinumab/Stelara, now considered as the reference product (ustekinumab RP) with the emergence of biosimilars. Demographic, clinical characteristics and outcomes are described for patients with IBD from the UVA IBD clinic who were identified to have experienced infusion reactions to ustekinumab and were found to have serum IgE specific for α‐gal. Patient charts were reviewed with approval from the UVA Health System Institutional Review Board (IRB). Patient consent was not required as part of this retrospective chart review.

To determine whether ustekinumab expresses α‐gal that would be accessible to IgE antibodies, an ImmunoCAP‐based assay (Thermo‐Fisher/Phadia, Waltham, MA, USA) was performed using serum from patients with established α‐gal syndrome (AGS) and non‐AGS controls. AGS was defined as a history of delayed allergic reaction to mammalian meat and detectable α‐gal IgE test. The FDA‐approved commercial α‐gal IgE assay utilizes beef thyroglobulin (BTG) on the solid phase. Patients and controls were recruited with the approval of the UVA Health System IRB, with all AGS patients and controls signing informed consent. Of note, serum samples were not available for those individuals with IBD who experienced reactions. In brief, ustekinumab (Stelara, Janssen Biotech Inc., Horsham, PA, USA) was biotinylated (EZ‐Link Sulfo‐NHS‐LC Kit, Pierce Biotechnology, Rockford, IL, USA) and 20 μg was conjugated to individual streptavidin ImmunoCAPs as the assay solid phase, as previously reported [9]. Cetuximab (Erbitux, ImClone LLC, Branchburg, NJ, USA) was biotinylated and 20 μg conjugated to individual ImmunoCAPs and run as a positive control. Given their role in IBD treatment, infliximab (Remicade, Janssen Biotech Inc., Horsham, PA, USA), which is also produced in SP2/0 cells, and vedolizumab (Entyvio, Takeda Pharmaceuticals USA Inc., Lexington, MA, USA), which is produced in CHO cells, were also prepared identically and assayed. For ustekinumab, IgE binding assays were carried out using two separate lots. All mAbs were conjugated using the same concentration and conditions. The amount of IgE against α‐gal that bound to each antigen was expressed in IU/ml (which equates to 2.4 ng/ml), as calculated by comparison to an internal heterologous standard curve, with a sensitivity cut‐off of 0.1 IU/ml. Assays were repeated in the presence or absence of  BTG, a competitive inhibitor that is heavily laden with α‐gal. BTG (Sigma‐Aldrich, St. Louis, MO, USA) was run at a saturating concentration of 2.5 mg/ml, as determined by preliminary experiments using BTG to block cetuximab.

As a functional assay, basophil activation tests (BAT) were conducted using peripheral blood from four patients with AGS or two non‐AGS controls with a commercial BAT kit (Bühlmann Diagnostics Corp., Amherst, NH), as previously described [21, 22, 23]. Basophil activation in response to ustekinumab was assessed across a range of concentrations identified in pilot studies (0.07 to 227 μg/ml) and compared to cetuximab (0.2 to 730 ng/ml) and infliximab (0.07 to 1136 μg/ml). Peripheral blood from two patients with AGS was also tested with vedolizumab. Flow cytometry was used to identify activated basophils as defined by CD63 upregulation of the CCR3 positive, Side Scatter (SSC) low population. Dose response curves were generated for each mAb and half‐maximal effective concentrations (EC50) calculated using a nonlinear regression and a three‐parameter response (GraphPad Prism Software [version 10.2.3 for Windows], Boston, MA) after data were normalized to the anti‐FceRI positive control (100%) and baseline negative control (stimulation buffer; 0%). Consistent with the manufacturer's instructions, all subjects were confirmed to have a positive response to the anti‐FceRI positive control that exceeded 15% of baseline activity. Alpha‐gal conjugated to human serum albumin (HSA) (Dextra, Reading, UK) was used in preliminary studies as an additional α‐gal control.

## 3 Results

Here, we describe six patients who had acute infusion reactions to ustekinumab, and, as part of follow‐up work‐up, were found to have IgE specific for α‐gal in their serum (Table 1). Five of these were ustekinumab naïve, whereas one patient had tolerated prior exposure to ustekinumab, but had been off treatment for over 18 months. Four patients had prior exposure to anti‐TNF agents. Urticaria was present in all cases, with some having systemic signs/symptoms. Levels of α‐gal IgE ranged from 1.88 to 13.6 IU/ml, with a mean value of 5.0 IU/ml. Details about food allergy history were only available in the chart for three cases, but among these cases none reported a history of red meat allergy despite regularly consuming red meat. Notably, all patients went on to tolerate subsequent subcutaneous administration. Some were pre‐treated with steroids and/or anti‐histamines prior to the first and second subcutaneous dose, but with additional experience patients received the first subcutaneous dose without pretreatment.

**TABLE 1:** Characteristics of patients who experienced infusion reactions to ustekinumab (Stelara) (UST) and tested positive for alpha‐gal IgE.

Age/sex and Crohn's montreal classification	Prior biologics	Prior UST exposure	Prior medication reaction	Description of UST reaction	Treatment	Pretreatment before first SC injection	Total IgE (IU/ml)	α‐gal IgE (IU/ml) (Ref < 0.1)
58 Female A2L3B2	Infliximab Adalimumab Vedolizumab Ustekinumab	Yes – (stopped due to insurance, 18 months prior)	No	Urticarial rash	Methylprednisolone Diphenhydramine Famotidine	Yes – pretreatment x2 injections	121	2.1
55 Male A3L1B2	Adalimumab	No	No	Urticarial rash	Methylprednisolone Diphenhydramine Famotidine	No	N/A	13.6
55 Male A3L3B2	Adalimumab	No	Yes—Adalimumab, papular dermatitis	Pruritus, Generalized erythema	Diphenhydramine	No	66	2.26
29 Male A2L1B1	Adalimumab Infliximab	No	Yes—Adalimumab, serum sickness	Urticarial rash, pruritus, flushing	Methylprednisolone Diphenhydramine Famotidine	Yes – pretreatment x2 injections	22.4	1.88
35 Male A2L3B2	No	No	No	Urticarial rash, Pruritus, Chest tightness	Methylprednisolone Diphenhydramine Famotidine	Yes – pretreatment cetirizine 10 mg prior	201	4.54
61 Female A3L2B1	No	No	No	Urticarial rash, pruritus, throat tightness	Methylprednisolone Diphenhydramine Famotidine	Yes—pretreatment cetirizine 10 mg prior	N/A	5.76

**FIGURE 1:**
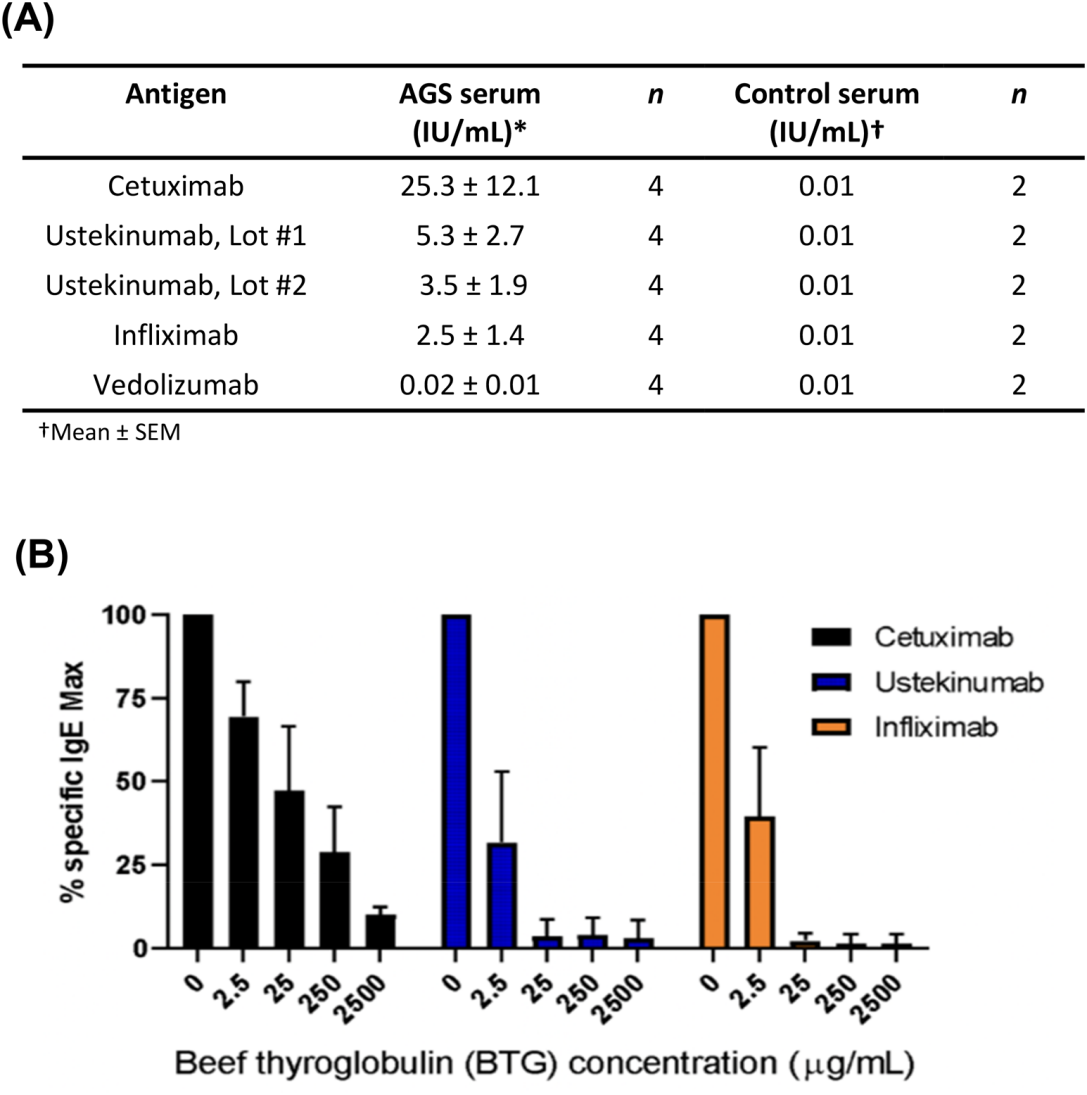
(A) IgE binding activity present in AGS patient or control serum to representative mAbs conjugated to the ImmunoCAP solid‐phase. (B) Competitive Inhibition Assay. Serum IgE specific for cetuximab, ustekinumab, and infliximab was measured in serum from AGS patients (*n* = 4), in the presence or absence of BTG. % specific Max expressed as mean +/− SD of the IgE value for each of the four subjects.

As expected, ImmunoCAP testing using serum from four patients with AGS revealed relatively high levels of IgE binding with cetuximab on the assay solid‐phase (mean 25.3 ± 12.1 IU/ml). Binding to ustekinumab was more moderate but was well above the assay threshold of 0.1 IU/ml and was similar for two separate lots of ustekinumab (Lot #1–5.3 ± 2.7 IU/ml and Lot #2–3.5 ± 1.9 IU/ml) (Figure 1A). Binding to infliximab was detectable at 2.5 ± 1.4 IU/ml. IgE binding to these three mAbs was markedly inhibited by competition with a separate α‐gal expressing glycoprotein, BTG (Figure 1B). Of note, even without BTG inhibition, IgE binding to vedolizumab was less than the assay cut‐off. Serum from two non‐AGS controls had no activity against any of the mAbs.

**FIGURE 2:**
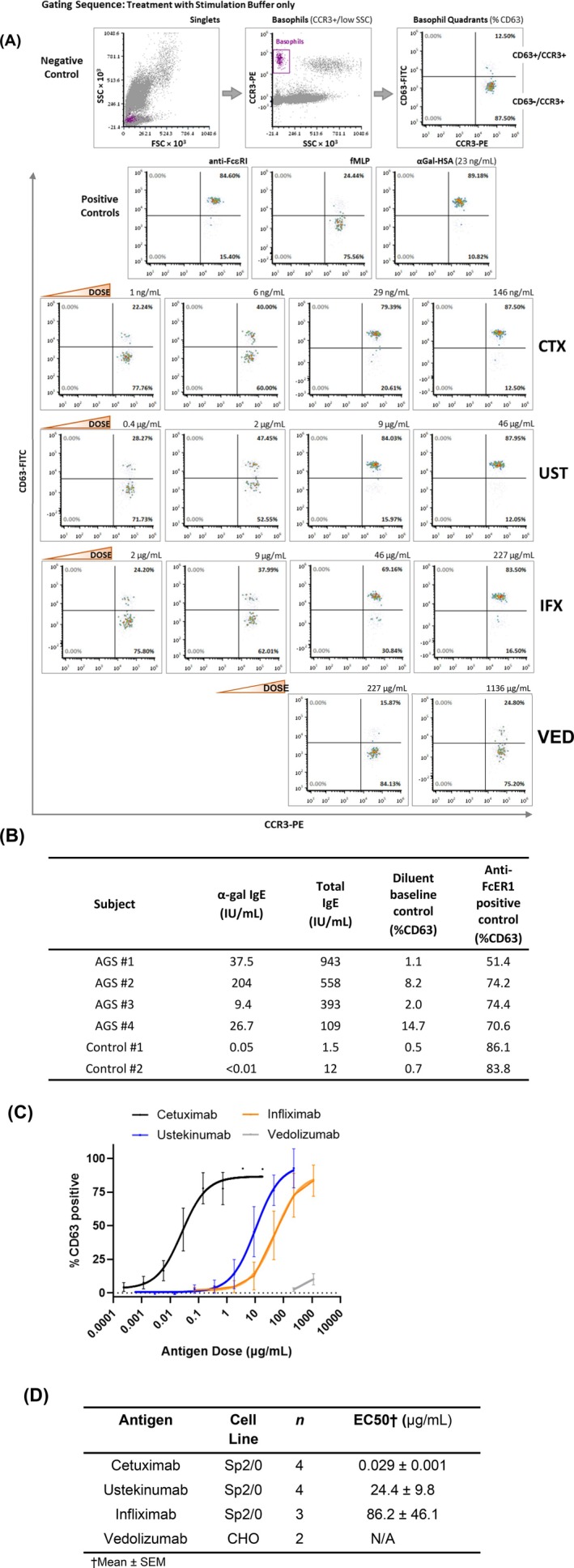
(A) Flow cytometry gating strategy and example of basophil activation testing (BAT). Flow data for singlet cells was plotted using Forward (FSC) versus Side (SSC) scatter and then as SSC versus CCR3‐PE. Data for cells within the basophil gate (CCR3+, SSC lo) was then plotted as CCR3 versus CD63‐FITC (the marker for activated basophils). The lower right quadrant shows quiescent basophils that are CD63‐/CCR3+. The shift of cells to the upper right quadrant (CD63+/CCR3+) indicates basophil activation. As expected, Positive Controls (anti‐FcεRI, fMLP, and alpha‐GAL‐HSA) showed increased frequency of CD63+ cells compared to the Negative Control (stimulation buffer alone). Dose response curves for Cetuximab (CTX), Ustekinumab (UST), and Infliximab (IFX) are shown with drug dose concentrations increasing from left to right; dot plots for two doses of Vedolizumab (VED) are also shown. (B) Characteristics of AGS and control basophil donors, confirming all subject basophils responded to the positive control. (C) BAT data from four AGS donors treated with varying concentrations of cetuximab, ustekinumab, infliximab, or vedolizumab, transformed in relation to positive and negative controls. Error bars reflect standard error of the mean. (D) Summary table of mAb activation In vitro BAT assay from AGS donors. CD63 expression was normalized to negative (buffer) and positive control (antiFcER1 IgG) for each sample assayed.

BAT was carried out on samples from four separate AGS patients as well as two non‐AGS controls (Figure 2A–D). The gating strategy is shown in Figure 2A. The mean response to anti‐FceRI positive control was 78.0% ± 5.3% (*n* = 6) whereas baseline activation (negative control) was 4.5% ± 2.3% (*n* = 6). When basophils were exposed to cetuximab, ustekinumab, or infliximab, all AGS patients had a dose‐dependent response in activation. Cetuximab was a more potent basophil activator than ustekinumab, which in turn had greater activity than infliximab. Infliximab activity was insufficient to calculate an EC50 for one patient, whereas vedolizumab (tested in two patients) had minimal activity even at the highest concentrations and an EC50 could not be determined. None of the mAbs had appreciable BAT activity when tested on cells from two healthy controls who were not α‐gal sensitized.

## 4 Conclusion

Our findings suggest that the presence of pre‐existing IgE antibodies specific to the oligosaccharide α‐gal is likely to be an unappreciated cause of some first‐dose hypersensitivity reactions to ustekinumab. While our results do not prove causality, the combination of clinical observation paired with laboratory investigation strongly suggests that α‐gal on ustekinumab has allergenic potential in α‐gal IgE sensitized individuals. The significance of this is bolstered by the fact that alpha‐gal IgE sensitization approaches or exceeds 20% in large parts of the United States [7, 13, 24]. Although not all of these cases met criteria for anaphylaxis, they were nonetheless severe enough to abort the infusion and require emergent medical care. Higher IgE binding and BAT potency for cetuximab versus ustekinumab may be due to the relative abundance of the glycan and/or to the established presence of α‐gal on the Fab region of cetuximab. When we embarked on this study evidence directly demonstrating that alpha‐gal was expressed on ustekinumab was lacking; however, it had already been reported that this monoclonal had only a single glycosylation site and that it was restricted to the Fc domain [25]. Recently, Cantin and colleagues confirmed alpha‐gal glycans on the Asn 299 site of the heavy chain [26]. Prior work supports the view that alpha‐gal restricted to the Fc domain is less allergenic than alpha‐gal expressed on the Fab, apparently related to steric availability [18, 25]. Notably, vedolizumab, which is produced in CHO cells, did not elicit significant IgE binding or basophil activation. This is consistent with data suggesting that mAbs produced in CHO generally do not have appreciable levels of α‐gal [9, 18].

These results highlight the importance of considering IgE to α‐gal as a potential mechanism underlying infusion reactions to ustekinumab and other biologics, such as infliximab, produced in SP2/0 cell lines [19, 20]. Notably, when administered subcutaneously, ustekinumab was successfully tolerated by all patients who had reacted to the initial intravenous infusion. Thus, for patients with mild to moderate reactions to intravenous ustekinumab, our data suggest that transitioning to the subcutaneous formulation can be done safely. In our experience, pre‐medication before subcutaneous injections is reasonable but not required; however, provision of an epinephrine autoinjector should be strongly considered in individuals who reacted to the intravenous infusion. With regard to differences in allergenic risk between intravenous versus subcutaneous administration, it could be a function of the route itself or of differences in dose administered. The intravenous loading dose for adults with inflammatory bowel disease is weight based and in the range of 260–520 mg, whereas for maintenance subcutaneous injections the dose is lower at 90 mg.

Limitations of this report include the relatively small size of the case series. The investigation of ustekinumab allergenicity was limited to ImmunoCAP and BAT‐based studies. Larger studies will be important to better understand the relative risk that ustekinumab presents among α‐gal sensitized individuals. In the case of cetuximab, studies have suggested that levels of pre‐existing α‐gal as low as or approaching 0.1 IU/ml can confer risk for reaction [11, 27, 28]. Given that ustekinumab is less allergenic than cetuximab in vitro (in relation to α‐gal), it is possible that only individuals with relatively higher α‐gal IgE levels would be at risk for reactions. To this point, in the cases we describe, the mean α‐gal IgE level was 5.0 IU/ml, with the lowest being 1.88 IU/ml.

Given the connection between α‐gal IgE and mammalian meat allergy, these data suggest a role for screening patients for a history of allergic reactions to mammalian meat prior to first infusion of ustekinumab. Inquiring about a tick bite history may also be informative as a strategy for minimizing risk given the well‐established link between pruritic tick bites and development of α‐gal IgE [13, 15]. In patients with a history of severe reactions to mammalian meat, consideration could be given to desensitization protocol for the initial infusion [29]. With the recent emergence of FDA‐approved ustekinumab biosimilars that are manufactured in CHO cells, there are also opportunities for head‐to‐head evaluation of allergenicity between versions of ustekinumab generated with SP2/0 and CHO cells. For example, ustekinumab‐auub (Wezlana, Amgen, Thousand Oaks, CA) and ustekinumab‐ttwe (Pyzchiva, Samsung Bioepis, Incheon, South Korea) are both produced in CHO cells [30]. We suggest further research in this space to better understand the relevance of α‐gal IgE in the allergenicity and risk related to ustekinumab and other biologics generated in mammalian cell systems. If allergenic risk to ustekinumab (Stelara) in AGS patients is confirmed in follow‐up investigations, selection of CHO‐produced biosimilars could be a practical way to mitigate risk in this patient population.

## Funding

This work was supported by National Institutes of Health, R37‐AI‐20565, R21‐AI166861.

## Ethics Statement

This work was approved by the University of Virginia Health System Institutional Review Board.

## Conflicts of Interest

T.P.M. and J.M.W. have received assay support from Thermo‐Fisher/Phadia for work unrelated to this project.

## Data Availability

The data that support the findings of this study are available from the corresponding author upon reasonable request.
